# Immersive high fidelity simulation of critically ill patients to study cognitive errors: a pilot study

**DOI:** 10.1186/s12909-017-0871-x

**Published:** 2017-02-08

**Authors:** Shivesh Prakash, Shailesh Bihari, Penelope Need, Cyle Sprick, Lambert Schuwirth

**Affiliations:** 1 Prideaux Centre for Research in Health Professions Education, Flinders University, Bedford Park, South Australia 5042 Australia; 2 Department of Intensive care, Flinders Medical Centre, 1 Flinders drive, Bedford Park, South Australia 5042 Australia; 3 Director of General Practice Training, Flinders Medical Centre, Flinders Drive, Bedford Park, SA 5042 Australia; 4 Simulation Unit, School of Medicine – Flinders University, Bedford Park, South Australia 5042 Australia; 5 Health Professions Education, School of Medicine, Flinders University, Bedford Park, South Australia 5042 Australia

**Keywords:** Cognitive bias, Decision-making, Diagnostic errors, Patient simulation, Physicians

## Abstract

**Background:**

The majority of human errors in healthcare originate from cognitive errors or biases. There is dearth of evidence around relative prevalence and significance of various cognitive errors amongst doctors in their first post-graduate year. This study was conducted with the objective of using high fidelity clinical simulation as a tool to study the relative occurrence of selected cognitive errors amongst doctors in their first post-graduate year.

**Methods:**

Intern simulation sessions on acute clinical problems, conducted in year 2014, were reviewed by two independent assessors with expertise in critical care. The occurrence of cognitive errors was identified using Likert scale based questionnaire and think-aloud technique. Teamwork and leadership skills were assessed using Ottawa Global Rating Scale.

**Results:**

The most prevalent cognitive errors included search satisfying (90%), followed by premature closure (PC) (78.6%), and anchoring (75.7%). The odds of occurrence of various cognitive errors did not change with time during internship, in contrast to teamwork and leadership skills (x^2^ = 11.9, *P* = 0.01). Anchoring appeared to be significantly associated with delay in diagnoses (*P* = 0.007) and occurrence of PC (*P* = 0.005). There was a negative association between occurrence of confirmation bias and the ability to make correct diagnosis (*P* = 0.05).

**Conclusions:**

Our study demonstrated a high prevalence of anchoring, premature closure, and search satisfying amongst doctors in their first post-graduate year, using high fidelity simulation as a tool. The occurrence of selected cognitive errors impaired clinical performance and their prevalence did not change with time.

## Background

There has been increasing impetus worldwide on preventing medical errors, which have been estimated to be one of the leading contributors to poor outcome including mortality [1]. An important type of medical error concern diagnosis and clinical decision making, since they are highly prevalent, often preventable, and generally lead to greater morbidity and mortality than other types of error [2]. In fact, the majority of complaints brought against emergency physicians arise from delayed or missed diagnoses [3]. These errors in diagnosis and clinical decision making have been shown to arise due to technical/knowledge deficits, system related factors or cognitive errors. Of these cognitive errors have been shown to be major contributors (in more than three fourths of errors involving diagnosis and clinical decision making) [4, 5].

Cognitive errors may stem from inappropriate application or complete failure to apply knowledge or suboptimal ‘cognitive dispositions to respond’ (CDR) or biases [4, 6, 7]. The term CDR has been suggested to replace the term ‘bias’, to remove the negative connotations associated with the later [8]. These biases represent the manner in which a physician orientates and responds to the presenting complaints, symptoms and signs under circumstances of uncertainty [8]. Cognitive biases can prompt clinicians to make errors when pruning, selecting and/or validating a diagnosis resulting in missed/wrong diagnoses and treatment [6, 9–15]. There are several factors which predispose to cognitive errors such as fatigue and sleep deprivation, affective state, patient factors, ambient conditions and past experiences [8].

The cognitive errors have been studied extensively in high-stakes safety industry such as aviation [16, 17]. However, in the medical literature the focus has been relatively recent [6, 18–22]. Also, the evidence on cognitive errors largely originates from studies on speciality trainees, and reveal that practicing physicians and trainees demonstrate considerable susceptibility to various cognitive errors. The initial post graduate years involve a sharp learning curve [23], wherein foundations of their non-technical and technical skills are laid with implications on their future performance. Therefore, identifying the common cognitive errors and how they impact performance can help prioritising them for focused interventions [24, 25]. In the absence of a formal training in the current curriculum, clinical experience alone would have to be relied on to attenuate the harmful effects of cognitive errors. However, whether this is the case, and to what extent is unknown.

To study cognitive errors in medicine, doctors are usually made to engage in cognitive tasks of diagnosis synthesis and decision making under experimental conditions. The majority of studies have used case notes and low-fidelity computer screen-based simulations to engage doctors in these cognitive tasks. The degree of immersion, cognitive, physical and affective load imposed by widely used methods of case notes and computer screen-based simulations, is different from what doctors experience in real life setting [26, 27]. Although there is controversy as to the *learning* efficacy of low versus high-fidelity simulation; [28] for *research* studies on cognitive errors, the latter may be better suited [27, 29–31]. Hence, clinical problems, recreated in a high-fidelity simulated environment, with engineered complexity, uncertainty and temporal demands, is likely to reflect the real life challenges more closely. Observing and interviewing doctors who immerse and engage with patients and other staff in such an environment provides an opportunity for assessment of various technical and non-technical skills including diagnosis synthesis and decision making [32]. Occurrence of various cognitive errors and their impact on diagnosis synthesis and decision making can also be studied [25, 32], as various factors such as content knowledge, scenario complexity, environment, time of the day, experience etc. can be controlled to certain degree.

In this pilot study, we investigate the relative occurrence of selected cognitive errors amongst doctors in their first post-graduate year, using high fidelity clinical simulation as a tool.

Besides cognitive errors, the changes in teamwork and leadership skills were also assessed during the first post graduate year; as this is one of the main learning objectives of the existing simulation program.

## Methods

The study involved review of intern (PGY1) simulation sessions conducted in year 2014 at Flinders medical centre, South Australia. The study was approved by Southern Adelaide Clinical Human Research Ethics Committee (91.15).

### Simulation program

Simulation involved high-fidelity scenarios, using the SimMan simulator (Laerdal Medical, Wappingers Falls, NY, USA), and actors (trained simulation centre staff) representing other team members (surgeon, nurse, medical emergency registrar etc.). Each session started with briefing, followed by simulation and a debriefing session. Video assisted debriefing [CAE LearningSpace™ (CAE Healthcare, Canada)] was used with focus on technical skills, teamwork and leadership. The predominant debriefing style involved advocacy inquiry [33]. This involves pairing observations of participant’s actions (or inaction) with inquiry into participants’ thought processes during that moment.

The four simulation scenarios chosen for study were–(A) acute onset breathlessness in a post-operative knee surgery patient, secondary to cardiogenic pulmonary edema; (B) pain in abdomen and dizziness in a day 1 post hemi-colectomy patient who has deteriorating hypovolemic shock (covert to overt), secondary to intra-abdominal bleeding; (C) low-conscious state in a trauma patient secondary to narcotic overdose resulting from a prescription error, and (D) low-conscious state due to hypoglycaemia in a fasting diabetic patient awaiting surgery, where regular insulin dose was not withheld (Appendix). These scenarios were developed from actual incidents in hospital for learning purposes. In each quarter of internship year, the interns were exposed to one of the four scenarios. The sequence of exposure to these scenarios over the year was random. Each simulation scenario began with the nurse calling the doctor to see the unwell patient. Towards the end of session, the senior help (usually an ICU or admitting consultant) enters and requests a handover from the team.

Think-aloud: In think-aloud studies, people are asked to verbalize their thinking while performing tasks [34]. Researchers using this technique typically both observe and audiotape or videotape the participant [35]. Although it is often used, the technique is not entirely undisputed and there is some criticism in literature that thinking aloud and the limited capacity of memory may hinder the cognitive processes of the participant, thus affecting performance [36–38]. On the other hand, there is broad support for it as well, and it is often seen as the only available ‘next best thing’ to–the currently impossible–‘direct observation’ of thought processes [39]. An alteration to the conventional ‘think-aloud’ protocol is the use of ‘verbal probing techniques’ [40]. Immediately following a particular response, questions (probes), are asked to help reveal the thought process [41]. Investigators have previously found that asking post-process questions to subjects provided valuable information that made think aloud data easier to understand and interpret [42, 43].

The participants in our study were not given instructions beforehand to verbalize their thoughts. Instead their thought process was probed (prompts) by the patient, the nurse and the ‘senior help’ to help reveal their thoughts during simulation. Prompts by the patient were initiated when the participant appeared to have gathered initial patient data i.e. history and physical exam. Prompts such as “what’s wrong with me doctor?” and “what does that mean?” were used to get insight into working differentials and participant’s understanding of pathophysiology and synthesis from the clinical data. The prompts from the nurse involved “what are you thinking?” and “why do you think so?”. These prompts were initiated if the participant initiated treatments without verbalizing the diagnosis or clinical problem or the participant verbalizes the diagnosis without the basis for it. The processing framework for think-aloud data was similar to that described previously [44]. Verbal data was transcribed and broken down into smaller components by predefined codes such as signs, symptoms, action, treatment, labs, monitoring. Scripts were then analysed to ascertain relationship between codes and identification of inductive and deductive reasoning processes.

Towards the end of session, the senior help arrived and asked the participants to verbalize their assessment of the situation and actions, along with the basis for it. If the participants were not correct with the diagnosis, they were asked if there could be other differentials and what were the findings for and against each differential. By comparing responses against checklist of key expectations, this interview process allowed for gaining an *estimate* about their knowledge around pertinent diagnoses and management. For example, the participant was identified to have adequate knowledge if he/she was aware of bradypnoea, hypothermia and pin-point pupils, and use of naloxone as a part of narcotic toxodrome diagnosis and management respectively.

### Recording of cognitive errors

For pragmatic reasons, only selected cognitive errors (Table 1) were studied, of the more than 30 reported in literature [8]. The basis for selecting these was the prevalence as reported in previous studies [45], minimal overlap in theoretical construct, coverage of both diagnosis synthesis and treatment decisions and feasibility of studying them based on video review of simulation sessions. The simulation scenarios were developed to focus on technical skills (diagnosis and treatment) and non-technical skills of teamwork and leadership.

**Table 1:** Catalogue of cognitive errors used in this study [45]

1. Anchoring	Tendency to fixate on a specific feature of a presentation early in the diagnostic process at the expense of understanding whole situation.
2. Confirmation Bias	Seeking or acknowledging only information that confirms the desired or suspected diagnosis. As new information is available there is a tendency to select out information which supports initial hypothesis, rather than adjusting the initial hypothesis in the light of new information against the initial hypothesis.
3. Premature Closure	Accepting a diagnosis prematurely, failure to consider reasonable differential of possibilities. There is an obvious halt to the diagnostic process targeted at differentials.
4. Search satisfying	Once a diagnosis is made, there may be a tendency to stop searching for co-existing diagnoses or causes and complications of current diagnosis.
5. Commission bias	Tendency toward action rather than inaction. Performing un-indicated manoeuvres, deviating from protocol. May be due to overconfidence, desperation, or pressure from others.
6. Omission bias	Hesitation to start emergency manoeuvres for fear of being wrong or causing harm, tendency towards inaction.
7. Overconfidence	Tendency to act on incomplete information, intuitions or hunches. Too much faith is placed in opinion instead of carefully gathered evidence. Often reluctant accept suggestions to consider alternatives.

To identify the occurrence of cognitive errors, two investigators (SP and SB) independently analysed the video recording and ‘think aloud’ comments from participants [CAE LearningSpace™ (CAE Healthcare, Canada)]. The observations were recorded using a Likert scale based questionnaire tool (Table 2). This questionnaire was adapted from a previously published questionnaire [45], which was used in a similar context of simulated in-hospital acutely sick patient. The assessors, similar to our study, were trained and had expertise in the field. Also, the questionnaire was demonstrated to be reliable (Cronbach’s α 0.81). Both the investigators have expertise in the management of critically ill patients and underwent training at using this questionnaire, which involved (i) familiarising themselves with the theoretical constructs of cognitive errors and the questionnaire, (ii) going through published examples of such cognitive errors, (iii) observing 10 randomly selected simulation sessions and applying questionnaire and discussing differences if any and (iv) discussing how in each of the four study scenarios, the specific cognitive error may manifest. While both the investigators were aware of the main study objective of feasibility, one investigator (SB) was blinded to other research questions. The investigators indicated whether or not a particular cognitive error was made by a given participant using a five point Likert scale, with the following anchors: ‘Strongly disagree’; ‘Disagree’; ‘Neutral/Unsure’; ‘Agree’; ‘Strongly agree’. The theoretical construct of cognitive errors obtained from previous publication allowed for two items per error, without increasing the risk of redundancy, cognitive load and fatigue on the assessors, while still preserving the face validity. Anchoring was reflected by questions 2 and 9, commission by questions 3 and 10, confirmation by questions 6 and 13, overconfidence by questions 4 and 11, omission by questions 1 and 8, premature closure by questions 5 and 12, and search satisfying by questions 7 and 14. For each cognitive error, the scores were averaged to develop final score that reflected three final outcomes–‘Present’, ‘absent’ and ‘can’t be assessed or unsure’. A cognitive error was considered as either present or absent, if both the assessors agreed. The outcome was ‘unsure/can’t be assessed’ if marked by both the assessors as ‘unsure/can’t be assessed’ or there was difference in marking.

**Table 2:** Questionnaire tool to assess cognitive errors

1	Initiated critical treatments in a timely manner and at appropriate dose or extent
2	Does not allocate attention to all presenting issues
3	Treatments initiated which were not indicated based on established emergency procedures (desperation, “try anything”)
4	Requires prompting to call for help/resources or refuses to call upon prompting
5	Did not consider a thorough differential diagnosis
6	Tried to “make” new data/information fit a diagnosis
7	Once diagnosis is made searches for all causes of diagnosis. For eg. Does not stop searching for other fentanyl patches when one patch found.
8	Recognition of problem is promptly followed by intervention
9	Became focused on one issue at the expense of fully understanding the situation
10	Treatments initiated or actions undertaken which were not necessary
11	Initiated treatments at inappropriate dose without checking/confirming/consulting
12	Tendency to anchor on to salient feature early in the diagnosis process
13	Did not alter the diagnosis when hints were provided by nurse or patient to alternative diagnosis
14	Once diagnosis is made searches for all complications of the diagnosis

### Recording of non-technical skills

The non-technical skills were evaluated using previously validated, Ottawa Crisis Resource Management Global Rating Scale (Ottawa GRS) [46, 47]. The Ottawa GRS is divided into five categories of crisis resource management (CRM) skills based on recognized CRM literature: problem solving, situational awareness, leadership, resource utilization and communication. An overall rating category for CRM performance is also provided. Each category is measured on a seven-point anchored ordinal scale with descriptive anchors to provide guidelines on alternating points along the scale.

### Statistical methods

Both descriptive and inferential statistics were used. The results are presented using tables and graphs. Data is presented as median and Interquartile range (IQR), unless otherwise specified. Prevalence of cognitive errors is reported as a ratio of number of observed error type to total number of scenarios. Normality was assessed using Kolmogorov-Smirnov test. Associations between categorical and continuous variables were assessed using Chi-square test and Spearman’s Rho respectively. The relationship of delay in diagnosis and time spent in internship, with the occurrence of cognitive error, was studied after adjusting for scenario type, using generalized linear modelling and logistic regression. Time to diagnosis (dependent variable) across scenario types (factor variable) was compared using one way ANOVA. Trends in Ottawa GRS score over the PGY1 year was assessed using the Friedman test. The internal consistency and inter-rater agreement (for the final outcomes of ‘Present’, ‘absent’ and ‘can’t be assessed or unsure’) of questionnaire tool for assessment of cognitive errors was analysed using Cronbach’s alpha and Kappa statistic respectively. The thresholds for Cronbach’s alpha [48] and Kappa statistic [49] were 0.7 and 0.6 respectively. Analysis was performed using SPSS version 21 (IBM Corp., Armonk, NY, USA). For all tests, a two-sided *P* value of less than 0.05 was considered significant.

## Results

A total of 70 simulation sessions were reviewed over the year 2014. A total of 25 interns participated in these sessions as primary responder. There were 17 sessions each of cardiogenic pulmonary oedema, narcotic overdose and hypoglycaemia, and 19 sessions of haemorrhagic shock.

### Performance of questionnaire

The inter-rater agreement and internal consistency was good for all cognitive errors assessed, except ‘overconfidence’ (Cronbach’s alpha 0.46; Kappa statistic 0.17 for overconfidence).

### Overall prevalence of cognitive errors

**Fig. 1:**
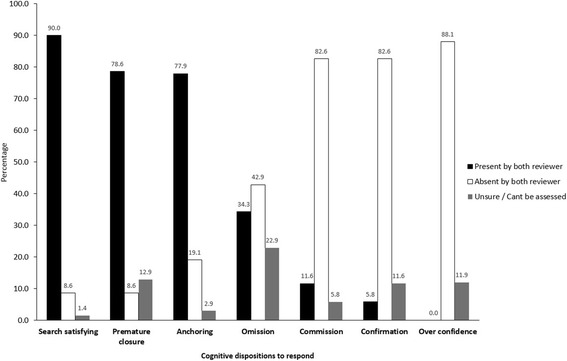
Overall prevalence of cognitive errors

Total number of studied cognitive errors observed per session ranged from 0 to 5, with a median (IQR) of 3 (2–4) errors per session. Frequent cognitive errors, observed in more than 75% of sessions, included search satisfying (90%), followed by premature closure (78.6%), and anchoring (75.7%) (Fig. 1). No instances of overconfidence were identified. The uncertainty was most frequent in the assessment of omission bias; whereas it was least frequent in the assessment of anchoring and search satisfying.

### Impact of occurrence of cognitive errors on diagnosis synthesis

The correct diagnosis was reached in 57 (85.1%) sessions, with a median (IQR) time of 4.05 (2.3–6) minutes. The scenarios differed in time to reach diagnosis (*P* = 0.04). After adjusting for the scenario type, anchoring appeared to be significantly associated with delay in diagnoses (β = 0.2, 95% CI 0.5 to 3.0, *P* = 0.007) and also occurrence of premature closure (x^2^ = 9.4, *P* = 0.005). There was a trend towards negative association between occurrence of confirmation bias and the ability to make a correct diagnosis (x^2^ = 5.8, *P* = 0.05).

### Occurrence of cognitive errors with time spent in internship

As shown in Table 3, there was no association of occurrence of particular cognitive error with scenario type, except for omission bias. After adjusting for scenario type, the odds of occurrence of various cognitive errors did not change with time spent in the internship year (Table 3).

**Table 3:** Association of cognitive errors with scenario types and odds of their occurrence with increasing internship time exposure

Cognitive error	Association with scenario type		Odds of occurrence with increasing internship time exposure	
	x^2^	*P* value	OR (95% CI)	*P* value
Search satisfying	0.47	0.9	1.2 (0.89;1.7)	0.21
Premature closure	2.0	0.57	0.75 (0.52;1.08)	0.12
Anchoring	2.4	0.4	0.84 (0.66;1.07)	0.15
Commission	3.4	0.3	0.99 (0.75;1.33)	0.91
Confirmation bias	2.2	0.52	1.02 (0.68;1.55)	0.91
Omission bias	10.3	0.02	1.3 (0.94;1.9)	0.11

### Other non-technical skills

**Fig. 2:**
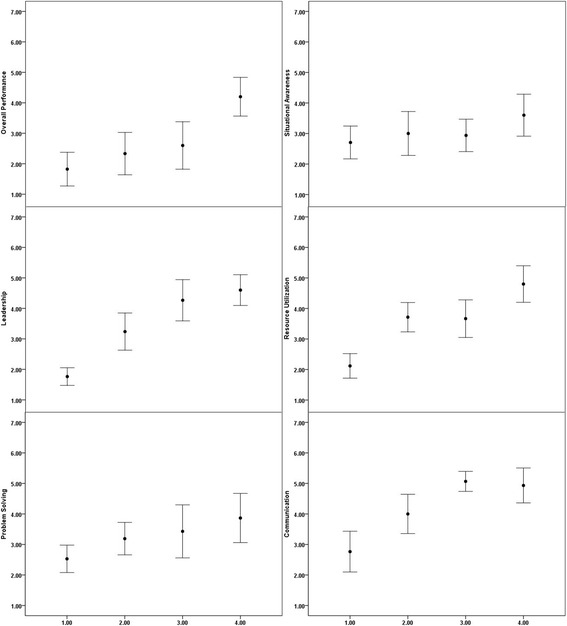
The Ottawa GRS score for non-technical skills across four quarters during the internship year

There was overall improvement of OTTAWA GRS scores during the internship period (x^2^ = 11.9, *P* = 0.01) (Fig. 2). As shown in Fig. 2, there was improvement in skills involving leadership (x^2^ = 28, *P* < 0.001), problem solving (x^2^ = 8.1, *P* = 0.04), resource utilization (x^2^ = 19.7, *P* < 0.001) and communication skills (x^2^ = 19.4, *p* < 0.001). No significant trend was seen in situational awareness (x^2^ = 1.9, *P* = 0.6).

Review of interview between ‘senior help’ and the participant revealed that although all participants had knowledge of clinical presentation of these diagnoses/syndromes, there were frequent deficits in the knowledge around management.

## Discussion

Our study shows that cognitive errors are highly prevalent amongst junior doctors in their first post-graduate year, particularly anchoring, premature closure and search satisfying. Although junior doctors’ clinical experience increased during the year leading to a significant improvement of their non-technical skills of teamwork and leadership, there was no change in the occurrence of studied cognitive errors. This is unfortunate because we also found that anchoring and confirmation bias did not only impact on the ability to make a diagnosis, but also resulted in a delay in making a diagnosis. Moreover, anchoring was associated with premature closure.

### Observed cognitive errors and their impact on diagnosis synthesis

The most frequent cognitive errors were ‘anchoring’, ‘premature closure’ and ‘search satisfying’. Anchoring was significantly associated with delay in reaching a correct diagnosis and premature closure. Anchoring represents the tendency to fixate on the hypothesis generated from a specific feature of a presentation early in the diagnostic process, at the expense of understanding the whole situation. Recovery from this initial anchor involves ‘adjusting’ to more appropriate diagnosis, by seeking additional data for and against the diagnostic hypothesis generated from the initial feature [50]. This is therefore likely to delay reaching correct diagnosis by the adjusting process [50], either rendered insufficient by premature closure, or flawed by confirmation bias. In confirmation bias, time is spent to recruit selective information to reinforce the initial impression that might have been generated due to anchoring; in which case, the correct diagnosis may not be even considered. The occurrence of confirmation bias becomes more likely when the information is presented sequentially, as is usually the case in clinical medicine [51]. This also reinforces the value of high-fidelity patient simulation for studying cognitive errors over case notes or computer screen-based tools, where the information is available upfront. The highly prevalent search satisfying by definition resulted in high incidence of failure to search for causes or contributing factors, complications and co-existing diagnoses. Attempts at identifying these are likely to result in more successful and effective management.

Overconfidence bias was not observed in our study. This is contrary to findings in the literature of considerable prevalence [12]. However, susceptibility to overconfidence bias may vary with seniority and may actually be low in fresh medical graduates, as supported by findings of Friedman et al. [52].

Despite the high prevalence of cognitive errors, the correct diagnosis was considered in majority of sessions. This is perhaps not as illogical as it seems: heuristics and biases that may underlie these errors, evolve over time to help perform under circumstances of uncertainty and high cognitive load, and hence they may not be always associated with poor outcome [53]. Also some of the cognitive errors studied may not impact diagnostic accuracy, but may influence the choices in management, eg. search satisfying may lead to missed co-diagnoses or complications. Increase in complexity of case, temporal demand or fatigue may uncover the potentially harmful effects of biases or cognitive dispositions. Besides these possibilities, it has also been shown that even if the correct diagnosis appears as one of the differentials, it is rarely rejected during the diagnostic process [54].

### High fidelity simulation as a research and teaching tool

Our pilot study is one of the first that demonstrates the feasibility of immersive high-fidelity clinical simulations to study cognitive errors, combined with a think aloud technique and a questionnaire based tool. This is a step forwards from previous studies utilizing predominantly case notes or computer screen based tools to study cognitive errors. Our study demonstrated the utility of simulation in eliciting how they may negatively impact clinical performance such as diagnosis synthesis and treatment decisions. Guided reflection here is likely to help develop catalogue of examples of situations where the potentially harmful effects of biases or cognitive dispositions may occur (exemplar theory [55]) and also motivate participants to learn (motivation theory [56]).

There was a significant improvement in teamwork and leadership skills. The efficacy of simulation based training for these skills have been demonstrated extensively in literature [57]. Similarly, focused simulation based training could help doctors to recognize and manage situations where they are at risk for cognitive errors. The applied psychology concepts, common to simulation based learning, of metacognition and ‘de-biasing’ strategies have been demonstrated in other fields to be effective in cognitive error prevention and recovery [58–61].

In the absence of formal training, clinical experience is often relied on to mitigate cognitive errors. In our study, 1 year of clinical experience did not appear to influence the occurrence of various cognitive errors. It is unlikely that the context of scenarios alone would have yielded this result as: (i) the sequence of scenarios were not fixed, (ii) the pattern of occurrence of cognitive errors across scenarios was largely similar and (iii) we adjusted for scenario type in the regression analysis. Since the study was not designed for this research question, the finding can only be regarded as hypothesis generating, to be tested in a larger prospective study. In support of this finding, however, is a prospective observation study which revealed that the clinical reasoning process remains relatively constant from medical school entry to practice [62]. Also, the high prevalence of cognitive errors amongst speciality trainees and practicing physicians suggests that experienced clinicians are as likely as junior colleagues to experience adverse cognitive dispositions or biases [63, 64]. There could be few reasons as to why clinical experience alone may not suffice as a remedy for cognitive errors. Firstly, the outcomes in real life may not be immediately visible and secondly, the learning that occurs from experience require unbiased and informed reflection (Kolb’s experiential theory) [65] on what cognitive errors are and how they can impair clinical performance. Observing clinicians during their work to enhance experiential learning around cognitive errors has ethical limitations as the error would need to be interrupted or ideally avoided, so that the patient is not harmed. Here simulation based learning may be beneficial as the errors can be allowed to evolve and serve as a learning catalyst.

### Resources involved in delivering high fidelity simulation based teaching

Simulation based teaching intervention can be resource intensive, and there is limited data in literature. Lapkin et al. reported that marginal cost of high fidelity simulation amounted to $ 291.26 per participant [66]. For our simulation based teaching, we used the resource cost model to estimate the cost [67]. There is one time cost of setting up of simulation facility which also includes purchase and installation of Mannequin, simulation software and computers. There are also recurring costs, of which the significant ones include equipment and materials, and personnel cost. Under our business model, the simulation based activity in the current project was a part of a larger simulation and clinical skills programme, used for training of medical students, post graduate doctors, nurses and allied staff. Hence the above costs which otherwise are significant (approximately $200,000) would be distributed across several teaching programs. Similarly, the cost involved in scenario development and programming was about $364.62 per scenario (6 h–$60.08/h). This cost again would be distributed across 30 to 40 participants each year, thereby significantly reducing the cost per participant. Per participant, the personnel cost involved in simulation based teaching was $157 (0.5 h nursing time, 0.6 h of 2 medical facilitators and 0.3 h technical and administrative time).

### Limitations and future research

We used retrospective study design which allowed for assessment of authentic performance of participants and their thought process using think-aloud transcripts. Although we were able to control for uncertainty, a prospective study using direct interview may help minimize uncertainty in assessment and also further validate the questionnaire based tool. The inter-rater agreement and internal consistency in assessment of Overconfidence was poor. This is likely to be highly skewed observations towards absence of Overconfidence.

Although, the assessors were well trained for making observations, there is no independent corroboration of assessor’s observations. Also only one of the two assessors was blinded to the research questions. Although this may have not affected the main objective of the study, it may impact on specific hypothesis testing. In future studies, it is essential that the assessors are blinded to the hypothesis being tested.

We used modified think-aloud technique using verbal probes. Although such technique allows for more focused information gathering, while minimizing interruptions, it can potentially introduce bias. Certain probe questions can be perceived as a cue to reconsider their thoughts/decisions. Despite their usefulness, unfortunately both conventional think-aloud technique and verbal probing have inherent shortcomings which are difficult to overcome [36–38]. Besides think aloud technique, there are other methods such as script concordance approach [68] and post-hoc cognitive walkthroughs [69] using video review, which may be trialled in future studies.

Cognitive errors were detected based on outcome measures, which could have been influenced by factors such as knowledge deficit. Since our assessment revealed deficits in knowledge around management of the simulated syndromes/diagnoses, some of these encountered errors may have originated from lack of knowledge, such as omission bias. This highlights the importance of assessment of the relevant content knowledge of participants, in order to better assess the origin of observed cognitive error. This is important to know, as interventions would differ. We did not have pre-defined standardized framework for assessment. Hence, although there was flexibility to modify assessment to the context, scenario and participant; this resulted in heterogeneity in assessment technique. A pre-formulated structured assessment framework will allow uniformity in assessment and needs to be trialled in a prospective study.

The results of our study need to be triangulated with a larger prospective study on cognitive errors using immersive high fidelity simulation as a tool. Also by controlling for various factors such as complexity of scenario, cognitive load, participant experience etc., and more objective assessment of participant knowledge base, it needs to be determined as to how and under what circumstances the cognitive dispositions or biases result in negative outcome. This is likely to provide essential evidence to facilitate studies into how simulation based education could be used to train doctors to avoid, identify and recover from cognitive errors.

## Conclusions

Our study demonstrated a high prevalence of anchoring, premature closure, and search satisfying amongst doctors in their 1st postgraduate year. The clinical experience gained during their first post graduate year and simulation based training focused on teamwork and leadership was associated with acquisition of these skills. However, there was no change in the prevalence of various cognitive errors. Further prospective research is needed to validate the results and explore the utility of simulation based intervention to help doctors manage their cognitive errors to improve clinical reasoning.

## Appendix

Details of scenarios with examples of cognitive errors.

### Scenario A: cardiogenic pulmonary edema

Clinical problem: Acute onset breathlessness in a post-operative knee surgery patient. Participant expected to manage acutely sick patient and work through differentials of sudden onset breathlessness.

Story and setting: The doctor is summoned by the bedside nurse to assess an elderly patient who is 3 days post knee replacement. He is in an orthopaedic ward, and develops sudden onset shortness of breath at rest. He has significant hypoxia and tachypnoea, which poorly responds to high oxygen delivery via non-rebreather mask. He is anxious, diaphoretic and cyanosed. He does not tolerate lying down. On auscultation the participant finds bilateral wheeze, basal crepts on lung auscultation and gallop rhythm on cardiac auscultation. He has cool peripheries but with strong pulse. He is in fast atrial fibrillation and is significantly hypertensive (BP systolic ~ 210 mmHg). There is no calf swelling/fever and operative site appears unremarkable, except for tenderness. He does not have chest pain but complains of tightness and difficulty breathing. He is not a diabetic, but does have a history of ischemic heart disease. He is one DVT prophylaxis and anti-platelet agents. During assessment, the patient at one instance coughs up pink frothy secretions.

Examples of cognitive traps:

**Table:**

CDR	Scenario specific examples
Anchoring	The participant displays a tendency to fixate on ECG changes/possibility of DVT/Bilateral wheezing and possibility of acute asthma early on in their workup.
Premature closure	Given the history of knee surgery, the participants commonly display premature closure with diagnosis of pulmonary embolism.
Search satisfying	Once recognizing the pattern of cardiogenic pulmonary edema, the participants either stop searching for likely causes or stop beyond the likelihood of ischemic cardiac event.
Confirmation	When thinking of pulmonary embolism, they display tendency to interpret post-operative knee pain as a feature of DVT and pink frothy secretions as ‘haemoptysis’ of pulmonary embolism. They then display tendency to ignore features against, such as orthopnoea, hypertension, and auscultation findings.
Overconfidence	Ignoring suggestion for calling for help by the nurse, despite deteriorating condition. Making guesses on medication dosage, despite available option of checking.
Commission	On deterioration, tendency to lie the patient down, despite marked orthopnoea. Tendency to commence IV fluid/administer fluid bolus, despite hypertension.
Omission	Despite, marked hypoxia, commencing oxygen delivery at very low rate (eg 2–4 L/min nasal specks) and then tendency to not escalate it further. Despite acknowledging very high blood pressure, not commencing treatment interventions.

### Scenario B: hypovolemic shock secondary to bleeding

Clinical problem: Pain in abdomen and dizziness in a day 1 post hemi-colectomy patient who has deteriorating hypovolemic shock (covert to overt) secondary to intra-abdominal bleeding.

Story and setting: The doctor is summoned by the bedside nurse to assess an elderly patient who is day 1, post hemi-colectomy and is complaining of abdominal pain poorly responding to ordered analgesics and dizziness. The patient is awake, anxious and diaphoretic. She complains of pain in the upper abdomen which is dull and poorly localized. The abdomen seems to be distended. The patient does have surgical drain which has poured out significant blood in the past 2 h, and also the patient has urinary catheter with poor urine output, but this information needs to be actively sought by the assessor (As the nurse is unaware of this). The patient’s blood pressure is normal initially but has sinus tachycardia, with cold peripheries and poor capillary refill. If the haemorrhagic shock is not recognized and managed appropriately, the patient’s condition deteriorates with narrowing of pulse pressure, followed by overt hypotension. In a completely neglected case, pulseless electrical activity ensues. If blood tests done, reveals low haemoglobin.

Examples of cognitive traps:

**Table:**

CDR	Scenario specific examples
Anchoring	The participants often anchor onto control of pain as the main issue. Also upper abdominal pain results in anchor into acute coronary syndrome. Some participants anchor onto abdominal distention with concerns of bowel distention and resort to placement of naso-gastric tube as a priority. All these examples involve failure to fully assess, gather all information and synthesizing the bigger picture. Another example would be recognizing low blood pressure and fixating on it using repeated fluid challenges, rather than working through the cause and treating it.
Premature closure	Post-operative ileus, myocardial infarction and analgesic management are examples of diagnoses, associated with failure to think through other possibilities.
Search satisfying	Once the haemorrhagic shock was recognized, there was failure to search for cause and complications. Example, efforts to look for anti-platelet agents or anti-coagulation, renal failure, hypothermia, medications causing low BP etc.
Confirmation	An example would be actively seeking abdominal x-ray and placement of nasogastric tube for abdominal distention, despite recognizing evolving shock and bleeding from the surgical drain.
Overconfidence	Ignoring suggestion for calling for help by the nurse, despite deteriorating condition. Making guesses on medication dosage, despite available option of checking.
Commission	Upon deteriorating using bag mask ventilation, despite patient breathing by herself. Some even elected to use GTN with the premature closure around myocardial infarction, despite the evolving shock.
Omission	Not commencing fluid bolus, or not giving fluid bolus beyond the initial bolus, or hesitancy in commencing bolus and only commencing slow infusion despite marked hypotension. Another example is not asking for blood transfusion despite noticing blood in the post-surgical drain.

### Scenario C: narcotic overdose

Clinical problem: low-conscious state in a trauma patient secondary to narcotic overdose resulting from prescription error.

Story and setting: The doctor is summoned by the nurse to assess this patient who is difficult to arouse. The patient was admitted last evening following a fall. He has bruise on his head and has fracture of his right wrist. He is due operation for his broken wrist later in the day. Last evening had a head CT which was normal (this information is available, if the participant asks for). He has two fentanyl patches which he applied himself before coming to hospital. Also there is duplication of opioid prescription (this information has to be actively sought by the participant, as the nurse is unaware). The patient has signs of opioid overdose–pinpoint pupils, low respiratory rate, low heart rate, and low body temperature. The participant is expected to work through causes of low conscious state, detect opioid toxodrome and treat accordingly.

Examples of cognitive traps:

**Table:**

CDR	Scenario specific examples
Anchoring	The participant had a tendency to anchor on the bruise and fixate on possibility of head injury. Other examples include anchoring on low sinus heart rate and focusing on workup for causes of sinus bradycardia, rather than looking at the bigger picture and synthesising the probability of narcotic overdose.
Premature closure	Premature closure was often seen around the diagnosis of head injury.
Search satisfying	Once opioid toxodrome was recognized, efforts were not made to identify the cause such as the additional fentanyl patch and duplication of opioid order. Other causes were not looked for/excluded such as electrolyte imbalance, hypoglycaemia other drug overdose.
Confirmation	Asking for another head CT, looking at the bruise. This was despite negative head CT 12 h back.
Overconfidence	Ignoring suggestion for calling for help by the nurse, despite deteriorating condition. Making guesses on medication dosage, despite available option of checking.
Commission	Upon deteriorating using bag mask ventilation, despite patient breathing by herself. Using atropine to increase heart rate. Using fluid bolus despite normal blood pressure.
Omission	Not using naloxone or hesitancy to re-administer or commence naloxone infusion.

### Scenario D: hypoglycaemia

Clinical problem: low-conscious state due to hypoglycaemia in a fasting diabetic patient awaiting surgery, where regular insulin dose was not withheld.

Story and setting: The doctor is summoned by the nurse to assess this elderly patient who is confused and more somnolent. The patient was admitted last evening following a knife injury to her hand. Overnight, she has been having pain issues, needing analgesics (including opioids). The patient is due tendon repair operation this morning and has been fasting for the same. Despite fasting, the patient got given insulin overnight (this info was not known to nurse and has to be sought by the participant). The patient is severely hypoglycaemic. The participant was expected to work through causes of low conscious state, detect hypoglycaemia and it’s cause and treat accordingly.

Examples of cognitive traps:

**Table:**

CDR	Scenario specific examples
Anchoring	The participant had a tendency to fixate on confusion in elderly in the setting of pain. Some had a tendency to fixate on opioid use and some on the nature of wound injury and possibility of sepsis.
Premature closure	Premature closure was often seen around the clinical syndromes of delirium, dementia, opioid toxodrome
Search satisfying	Once hypoglycaemia was detected, efforts were not made to find out the cause (prescription error) and hence prevention of further hypoglycaemia.
Confirmation	Despite no response to naloxone or no history of dementia and in a 12 h clean wound, persistent efforts to explore opioid, psychiatric and sepsis hypothesis
Overconfidence	Ignoring suggestion for calling for help by the nurse, despite deteriorating condition.
Commission	Upon deteriorating using bag mask ventilation, despite patient breathing by herself. Using fluid bolus despite normal blood pressure.
Omission	Despite noticing long acting insulin prescription, failure to hold that prescription and commencement of dextrose infusion to prevent recurrence.

## Abbreviations

CRM: Crisis resource management
GRS: Global rating scale
IQR: Interquartile range
